# Type I interferon pathway assays in studies of rheumatic and musculoskeletal diseases: a systematic literature review informing EULAR points to consider

**DOI:** 10.1136/rmdopen-2022-002876

**Published:** 2023-03-02

**Authors:** Agata Burska, Javier Rodríguez-Carrio, Robert Biesen, Willem A Dik, Maija-Leena Eloranta, Giulio Cavalli, Marianne Visser, Dimitrios T Boumpas, George Bertsias, Marie Wahren-Herlenius, Jan Rehwinkel, Marie-Louise Frémond, Mary K Crow, Lars Ronnblom, PG Conaghan, Marjan Versnel, Ed Vital

**Affiliations:** 1 Leeds Institute of Rheumatic and Musculoskeletal Medicine, University of Leeds & NIHR Leeds Biomedical Research Centre, Leeds, UK; 2 University of Oviedo, Area of Immunology, Instituto de Investigación Sanitaria del Principado de Asturias (ISPA), Oviedo, Spain; 3 Charité University Medicine Berlin, Department of Rheumatology, Berlin, Germany; 4 Erasmus MC, University Medical Center Rotterdam, Laboratory Medical Immunology, Department of Immunology, Rotterdam, Netherlands Immunology, Rotterdam, The Netherlands; 5 Uppsala University, Department of Medical Sciences, Rheumatology, Uppsala, Sweden; 6 Unit of Immunology, Rheumatology, Allergy and Rare Diseases, Vita-Salute San Raffaele University, Milan, Italy; 7 EULAR, PARE Patient Research Partners, Amsterdam, Netherlands; 8 University of Crete, Medical School, Department of Internal Medicine, Heraklion, Greece; 9 University of Crete, Medical School, Department of Rheumatology-Clinical Immunology, Heraklion, Greece; 10 Karolinska Institutet, Division of Rheumatology, Stockholm, Sweden; 11 Broegelmann Research Laboratory, Department of Clinical Science, University of Bergen, Norway; 12 Medical Research Council Human Immunology Unit, Medical Research Council Weatherall Institute of Molecular Medicine, Radcliffe Department of Medicine, University of Oxford, UK; 13 Université de Paris Cité, Hôpital Necker-Enfants Malades, Immuno-Hématologie et Rhumatologie pédiatriques, Paris, France; 14 Hospital for Special Surgery, Weill Cornell Medical College, Mary Kirkland Center for Lupus Research, New York, USA; 15 Erasmus MC, Department of Immunology, Rotterdam, The Netherlands

**Keywords:** Arthritis, Rheumatoid, Cytokines, Inflammation, Lupus Erythematosus, Systemic

## Abstract

**Objectives:**

To systematically review the literature for assay methods that aim to evaluate type I interferon (IFN-I) pathway activation and to harmonise-related terminology.

**Methods:**

Three databases were searched for reports of IFN-I and rheumatic musculoskeletal diseases. Information about the performance metrics of assays measuring IFN-I and measures of truth were extracted and summarised. A EULAR task force panel assessed feasibility and developed consensus terminology.

**Results:**

Of 10 037 abstracts, 276 fulfilled eligibility criteria for data extraction. Some reported more than one technique to measure IFN-I pathway activation. Hence, 276 papers generated data on 412 methods. IFN-I pathway activation was measured using: qPCR (n=121), immunoassays (n=101), microarray (n=69), reporter cell assay (n=38), DNA methylation (n=14), flow cytometry (n=14), cytopathic effect assay (n=11), RNA sequencing (n=9), plaque reduction assay (n=8), Nanostring (n=5), bisulphite sequencing (n=3). Principles of each assay are summarised for content validity. Concurrent validity (correlation with other IFN assays) was presented for n=150/412 assays. Reliability data were variable and provided for 13 assays. Gene expression and immunoassays were considered most feasible. Consensus terminology to define different aspects of IFN-I research and practice was produced.

**Conclusions:**

Diverse methods have been reported as IFN-I assays and these differ in what elements or aspects of IFN-I pathway activation they measure and how. No ‘gold standard’ represents the entirety of the IFN pathway, some may not be specific for IFN-I. Data on reliability or comparing assays were limited, and feasibility is a challenge for many assays. Consensus terminology should improve consistency of reporting.

**WHAT IS ALREADY KNOWN ON THIS TOPIC**

- Type I and type II interferons play a role in a broad spectrum of rheumatic musculoskeletal diseases (RMDs).
- There is a large body of literature indicating that many different assay methodologies evaluating distinct steps of type I interferon (IFN-I) pathway activity may have roles in the diagnosis, prognosis, therapy selection and stratification for therapy in RMD patients. However, no consensus on the best method has been proposed.

**WHAT THIS STUDY ADDS**

- This study provides a systemic literature review and synthesis of all published data on IFN-I assays reported in basic and clinical research in RMDs, especially the most substantial literature on gene expression and protein assays in systemic lupus erythematosus.
- We provide an appraisal and commentary of an expert group on content and criterion validity, reliability and feasibility of these assays.
- We also propose consensus terminology for future IFN assay reporting in basic and clinical research.

**HOW THIS STUDY MIGHT AFFECT RESEARCH, PRACTICE OR POLICY**

- This work will assist physicians and researchers to select the most appropriate assays for the analysis of IFN-I pathway activity and facilitate the translation of IFN-I pathway activation assays into clinical practice.

## Introduction

Type I interferons (IFN-Is) are a group of cytokines with antiviral and immune-modulatory function. IFN-Is have roles in several rheumatic musculoskeletal diseases (RMDs), Many IFN-I assays have been proposed to aid in clinical management of RMDs but, despite a large body of literature, not yet adopted into routine clinical practice.^1^

The IFN-I family comprises 13 functional IFN-α genes and one IFN-β gene on human chromosome 9, as well as IFN-ε, IFN-κ, IFN-τ and IFN-ω. IFN-I subtypes are produced by all nucleated cells. In acute viral infection circulating haematopoietic immune cells, especially plasmacytoid dendritic cells, are the most important producers. In other contexts production by stromal and parenchymal cells in most tissues may be more important.^2 3^ These proteins bind to a shared receptor (IFNAR), initiating a cascade of downstream molecular and cellular effects. Signalling via the JAK-STAT pathway leads to expression of IFN-stimulated genes (ISG) that contain the IFN-sensitive response element. These ISGs encode intracellular, surface and soluble proteins that have diverse effects on immune regulation and on antiviral response, with significant remodelling of mRNA processing, post-translational modifications, metabolism, cellular trafficking, chromatin organisation and the cytoskeleton, among others.^4 5^ Although there are distinctions in the signalling pathway and response elements between type I, type II (IFN-γ) and type III IFNs (IFN-λs), there is also considerable overlap between these systems which may make interpretation of these downstream pathways difficult.^6^ ISG expression on similar stimuli may differ between cell types and tissues.^1^

IFN-I have pathogenic roles in a broad range of human diseases including autoimmunity, infection, cancer and cardiovascular disease.^7 8^ In RMDs, IFN-I acts as a mediator linking innate and adaptive immunity, with special significance in diseases in which self-nucleic acids are sensed by IFN-producing innate immune cells, in which they probably promotes the production of antinuclear antibodies.^9 10^ Therapeutic monoclonal antibody blockade of IFNAR was effective in phase III clinical trials in systemic lupus erythematosus (SLE), and is being investigated in other RMDs, and IFN-I pathway blockade may be involved in the mechanism of action of other therapies such as anti-malarials and JAK inhibitors.^11 12^

Assays for IFN-I have been proposed to have roles in the diagnosis, prognosis, therapy selection and stratification for therapy in RMD patients, to reclassify RMDs, as well as predict disease onset.^13^ A limitation in the progression of these assays into routine practice has been the number and heterogeneity of methods and clinical studies published.^1^ Assays include methods to measure IFN-I proteins,^14^ gene expression assays for ISGs, assays for proteins encoded by ISGs, DNA methylation and functional assays. These are paralleled by heterogeneity in the diseases studied, clinical questions addressed and design of clinical studies. As a result, there has been no consensus on the type of IFN-I assay that should be used, nor in what clinical indications. An additional issue is the use of varying, and sometimes contradictory, terminology to refer to aspects of the biological pathway and systems for evaluating it.

To address these issues, a EULAR task force was convened. We aimed to conduct a systematic literature review (SLR) on the principles and performance metrics of the assays described in the field of RMDs and to develop consensus terminology for use in future studies.

## Methods

EULAR standardised operating procedures for EULAR-endorsed recommendations were followed.^15^ A multidisciplinary task force of 17 members (from 8 EULAR countries and the USA) was convened including experts in all techniques used for IFN-I pathway activation assays, as well as autoimmune rheumatic disease, viral immunology and monogenic interferonopathies. The task force included an expert in EULAR methodology, two members of the EULAR emerging network (EMEUNET) and a patient representative. Six Population Intervention Comparator and Outcome questions (PICOs) were formulated (online supplemental text 1). PICO 1, ‘What methods have been employed to assess type I IFN pathway activation in people with RMDs? What are their performance metrics (including aspects of content, criterion, construct validity as well as reliability and feasibility) of these methods?’ is the basis of this SLR. PICOs 2–6 refer to clinical applications and will be reported separately (reference to SLR2) (online supplemental text 1).

10.1136/rmdopen-2022-002876.supp1 Supplementary data

### Search strategy and eligibility criteria

A protocol for the SLR was developed and approved by the task force. Ovid Medline, Embase and Web of Science were searched for reports of IFN and RMDs up to October 2019. Search strategy is provided in online supplemental text 2–4. In addition to the RMD terms, papers were eligible for inclusion if they fulfilled the following criteria: (1) presented data on human patients with RMDs (with or without healthy controls); (2) design as cross-sectional; randomised control trials, case–control studies, non-controlled trials, diagnostic accuracy studies, cohort studies, intervention studies; (3) studies that described results on biological material derived from peripheral blood (ie, serum, whole blood, cell subsets); (4) written in English. Exclusion criteria were: (1) non-human studies; (2) conference abstracts, case studies, non-original articles such as editorials, review, opinion pieces, (3) articles that did not specify the type of IFN that the assay measures; (4) papers that did not describe their results as an assay, biomarker, test, score or similar in the abstract, (5) studies purely on IFN-I pathway genetics (see online supplemental text 5 for details). A minimum sample size was not considered within eligibility criteria as it could hid studies with more complex methods.

Titles and abstracts, followed by full-text screening was performed by two reviewers (AB and JR-C). The agreement between reviewers was high (>95%) and discrepancies were resolved by discussion or consultation with the convenor (EV).

### Data extraction

An extraction template addressing all PICOs was developed. For the present report, the following fields were collected: method name, population studied, type of assay, material analysed, pathway element, detailed description of method and calculation of reported result, validity (including face validity, criterion validity, concurrent validity, discussed further below), reliability and feasibility. Association with clinical endpoints (ie, diagnostic accuracy) as well as assay responsiveness were analysed in a separate SLR (reference to SLR2). Due to the heterogeneity in methods and analyses reported, comparative statistical analysis or meta-analysis was not performed, and the results are presented in narrative form.

### Interpretation of IFN assay validity

All assays included in SLR met face validity since the literature review filter implied a plausible role in the IFN pathway. For content validity, we described what aspects of the IFN pathway are measured in a description of principle of the assay. For criterion validity, no objective gold standard type I IFN pathway activation assay exists. We therefore described criterion validity as ‘evidence that assay measures IFN-I’ and four options were considered: (1) experimental stimulation of cells with IFN-I in vitro and demonstration of assay induction, (2) assays in patients receiving IFN-I therapy, (3) standard curves where appropriate, for example for an IFN-alpha ELISA but not IFN-inducible chemokines, (4) blocking with anti-IFN antibodies in vitro or as a therapeutic agent. For concurrent validity, we evaluated whether putative IFN-I assays were shown to correlate with other putative assay(s). For reliability, we sought evidence of reanalysis of samples in independent laboratories by the same method or repeat analysis of a sample at different times. Information on feasibility was provided based on review of the paper methodologies by the TaskForce panel as well as review of data in the paper.

### Development of consensus terminology

After review of extracted data, terminology to describe aspects of type I IFN pathway, and assays designed to measure it, was developed by the task force. First, common themes with ambiguous concepts or that require harmonisation were identified. Next, definitions were produced by an iterative process until consensus was achieved.

## Results

### Summary of RMDs and assays reported in the literature

**Figure 1:**
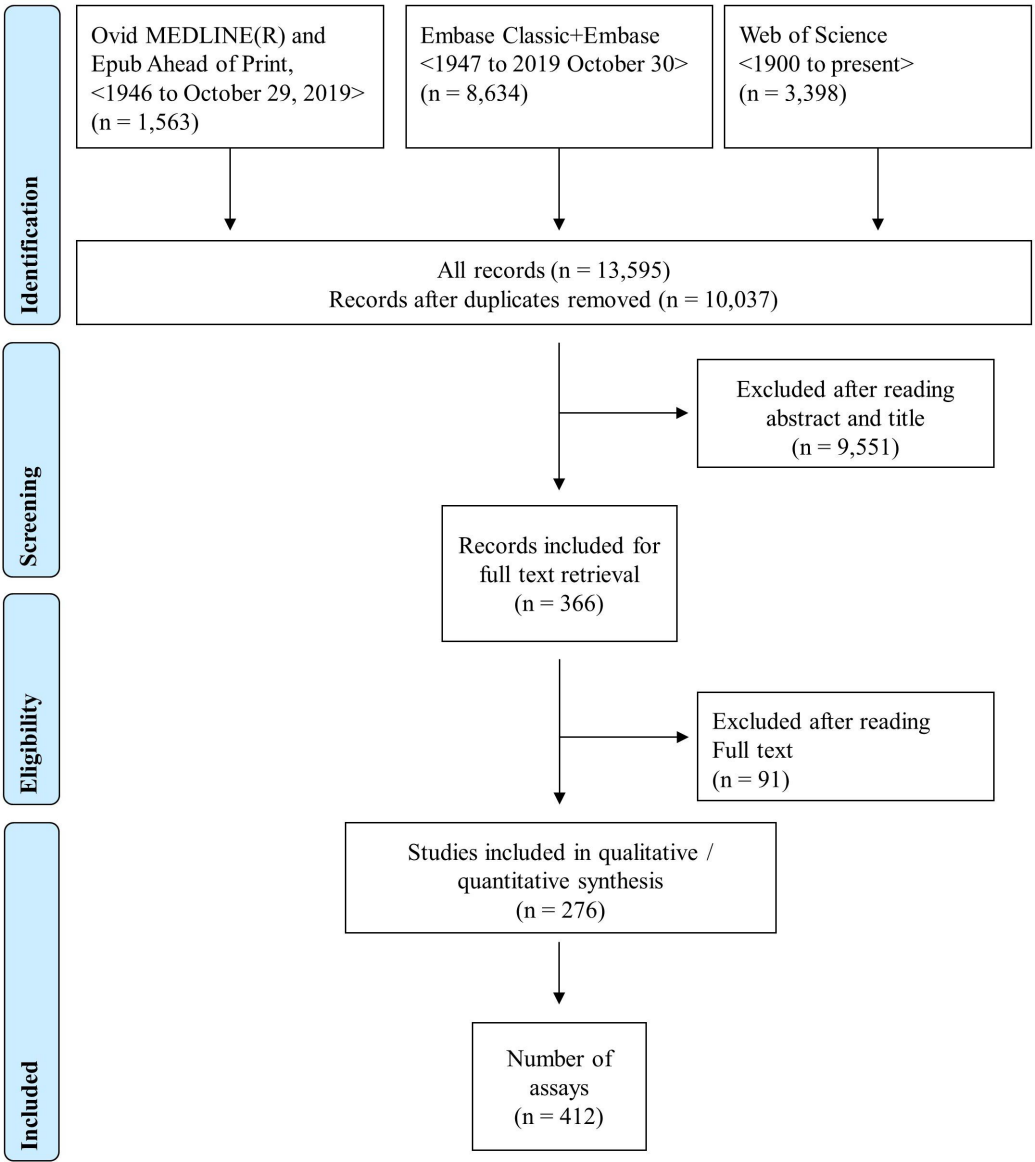
PRISMA chart. Search and selection strategy of publications. PRISMA, Preferred Reporting Items for Systematic Reviews and Meta-Analyses.

Study selection is summarised in the Preferred Reporting Items for Systematic Reviews and Meta-Analyses diagram (figure 1). A total of 10 037 abstracts were identified. A total of 276 of these reports fulfilled eligibility criteria and were used for data extraction. Some used more than one technique to measure IFN-I pathway activation. Hence, these 276 papers generated data on 412 methods.

**Figure 2:**
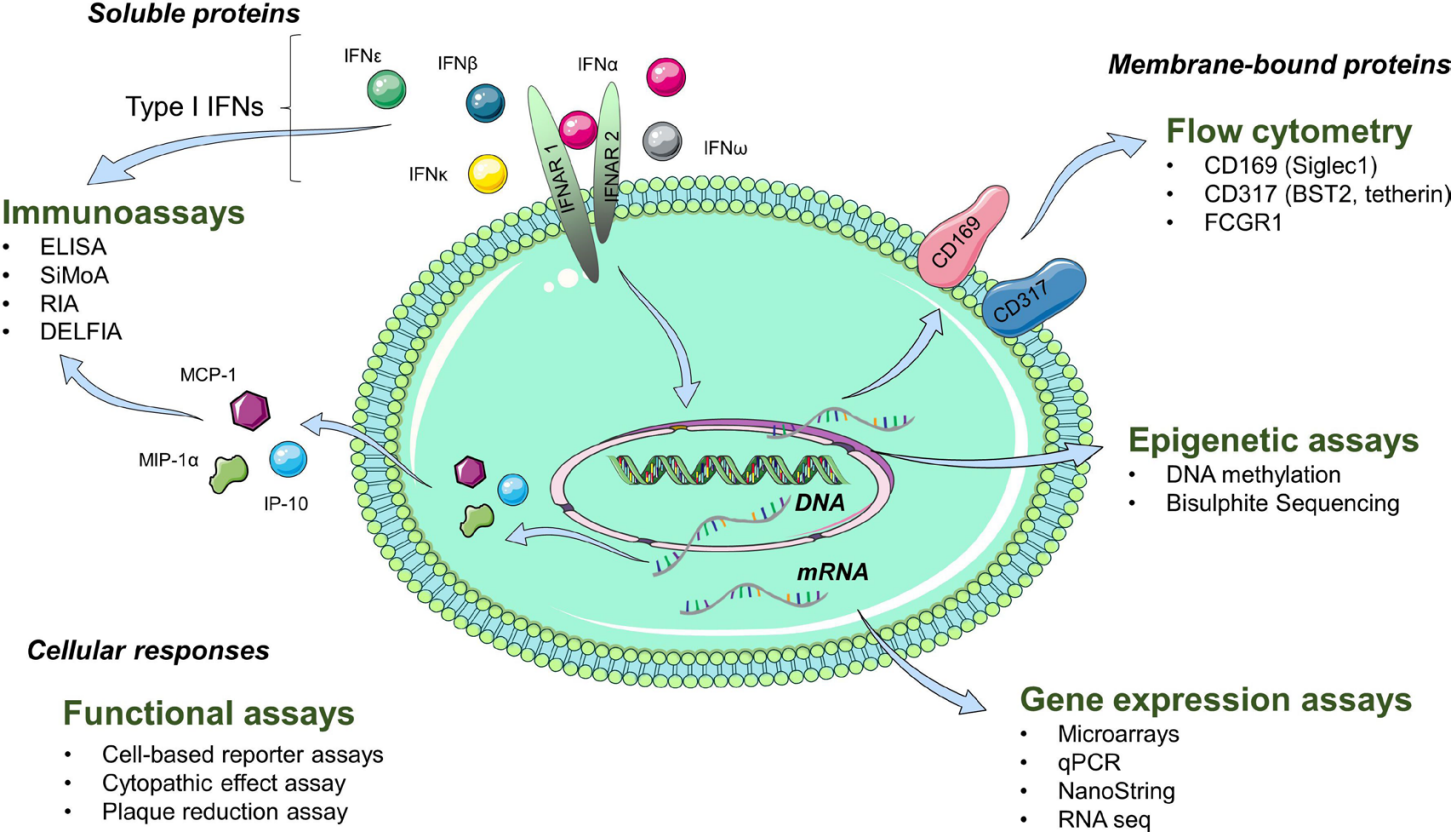
Aspects of the IFN-I pathway evaluated by each assay. The IFN pathway is a complex system with multiple subtypes of IFNs and diverse downstream effects on gene and protein expression. Existing assays measure different aspects of the IFN pathway; they do not reflect the entirety of the pathway and some are not specific for IFN-I. See text for full description of these assays.

**Figure 3:**
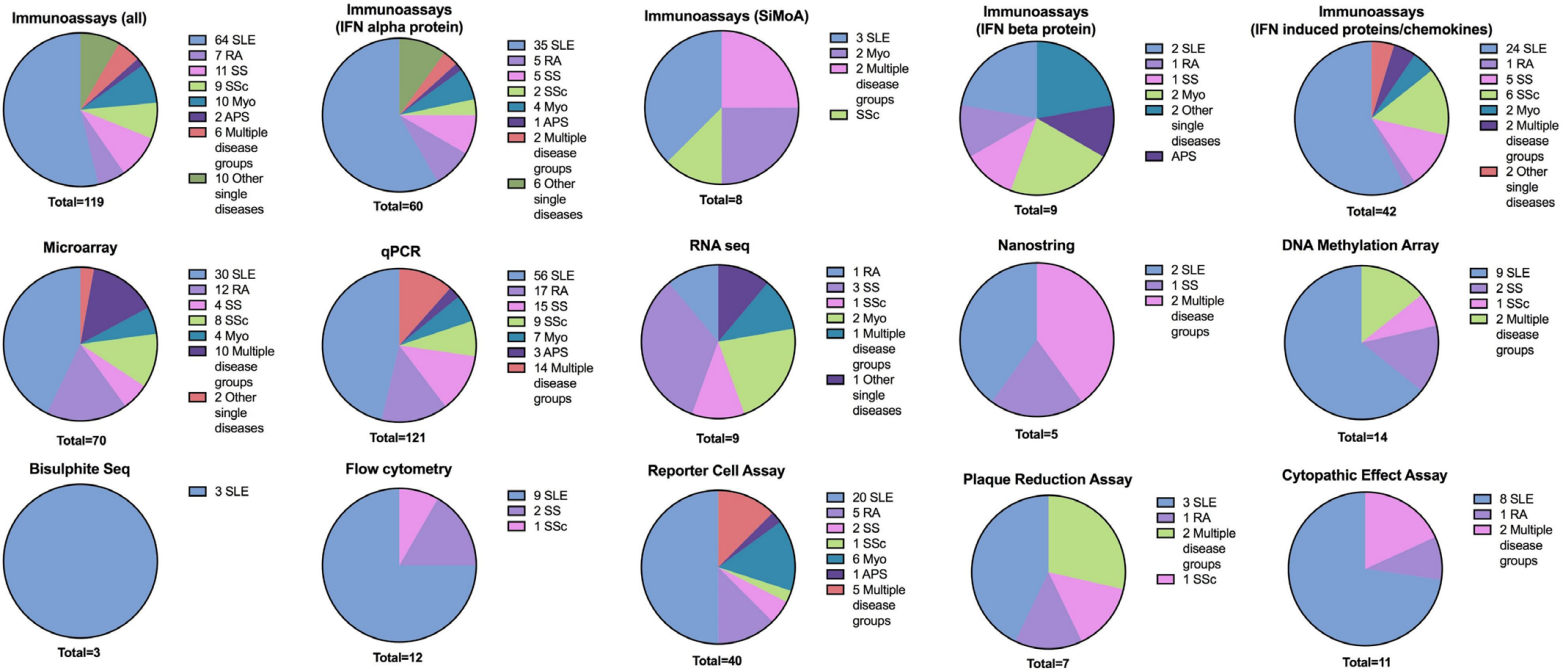
Summary of RMDs evaluated using each type of IFN-I assay. Pie charts indicate the number of reports of assays for each methodology and each rheumatic musculoskeletal disease. Some publications include more than one assay, so number of papers may differ from the numbers on this figure. RMDs, rheumatic musculoskeletal diseases.

Assays measured diverse aspects of the IFN-I pathway (figure 2). A summary of the assays used and their classification is in table 1. The most frequently studied RMD was SLE (n=204 reports), followed by RA (n=43), SS/pSS (n=41), SSc (n=32), myositis (n=29), antiphospholipid syndrome (APS) (n=6), multiple disease groups (n=44), other single RMDs such as AS, PsA, AAV, Behçet's disease, IgG4-RD (n=13). Sample sizes ranged from n=6 to n=1760.^16 17^ Types of assays also varied across RMDs (figure 3).

**Table 1:** Summary of IFN-I assays reported in the literature

	Category	Name	What it measures	No of studies	Validation that assay measures IFN-I (n/N)	Evidence of concurrent validity (n/N)	Quantitative or categorical	Ref
1.	Immunoassay	Immunoassays for IFN -α protein	IFN-α protein	60	58/58	12/57	Quantitative	18–26 46 47 57–103
		SiMoA	IFNs or chemokines	8	6/8	5/8	Quantitative	3 27 28 38 39 104 105
		Immunoassays for IFN -β protein	IFN -β protein	9	9/9	4/9	Quantitative	16 18 20 66 73 82 95 105 106
		Immunoassays for IFN-inducible proteins	IFN-inducible soluble proteins	42	3/42	16/42	Quantitative	21 22 26 29–40 61 62 74 77 78 83 103 106–125
2	Flow cytometry	Flow cytometry	IFN inducible surface markers	12	4/12	5/12	Quantitative	22 35 39 41 43 70 77 120 121 126–128
3	Microarrays	Microarray modules	Subsets of ISGs (modules)	10	1/10	4/10	Either	38 113 118 129–135
		Microarray scores	Selected gene analysis	25	8/25	14/25	Quantitative	20 25 29 37 40 112 125 133 135–150
		Microarray signatures	Whole transcriptome unsupervised analysis	35	7/35	14/35	Categorical	17 34 43 62 70 82 127 151–178
4.1	qPCR	pPCR for IFNs	IFN-α or β gene expression	4	1/4	1/4	Quantitative	57 89 98
		qPCR: individual ISGs	Individual ISG expression	30	4/30	15/30	Quantitative	57 65 129 137 139 144 179–187
		qPCR IFN scores	Expression of a set of ISGs	82	8/82	25/82	Quantitative or categorical	3 13 27 28 32–35 41 48–54 105 109 121 125 136 137 145 157 161 166 168 171 173 188–237
		qPCR Chemokines	IFN induced chemokines	4	1/4	2/4	Quantitative	201–203 238
		qPCR ISG clusters	ISG signature	2	0/2	0/2	Categorical	153 170
5	RNASeq	RNASeq	Whole transcriptome analysis	9	3/9	3/9	Categorical	28 44–46 105 116 211 239 240
6	Nanostring	Nanostring	Expression of a set of ISGs	5	1/5	4/5	Quantitative	17 28 39 46 47
7	DNA methylation	DNA methylation	ISGs in whole genome DNA methylation arrays	14	0/14	10/14	Categorical	45 125 181 241–251
8	Bisulphite sequencing	Bisulphite sequencing	Validation for genes from DNA methylation studies	3	1/3	0/3	Categorical	244 246 252
9.1	Reporter cell assay	Reporter cell qPCR	Cell line or PBMCs) stimulated with patient sample then ISG qPCR	31	14/31	8/31	Quantitative	30 32 40 65 82 182 204 212 229 253–273
9.2	Reporter cell assay	Reporter cell other	Cell line or PBMCs stimulated with patient sample then luminometric or colorimetric methods	9	2/9	5/9	Quantitative	20 24 137 149 163 274–276
10	Cytopathic effect assay	Cytopathic effect assay	Tests whether the functional effect of IFNs in protecting cells from viral infection can be observed by cytopathic features in cultured cells	11	9/11	3/11	Semiquantitative	3 23 27 277–284
11	Plaque reduction assay	Plaque reduction assay	Tests whether the functional effect of IFNs in protecting cells from viral infection can be observed by size of plaque of cultured cells	7	7/7	0/7	Semiquantitative	42 285–290
12	IHC	IHC in whole blood	Whole blood stained for MxA	1	0/1	0/1	Semiquantitative	122

IHQ, Immunohistochemistry; ISG, IFN-stimulated genes; PBMCs, Peripheral Blood Mononuclear Cells.

### Description of assays reported in the literature

A detailed description of the principles of each assay is given in online supplemental text 6.

### Immunoassays: IFN-I proteins (IFN-α and IFN-β)

Fifty-eight studies were identified for IFN-α and nine for IFN-β.

Content: Antibody-based methods to quantify IFN-I proteins in biosamples including ELISA, dissociation-enhanced lanthanide fluorescence immunoassay (DELFIA), multiplex assays and radioimmunoassays (RIA).

Criterion validity: These assays use specific antibodies, mostly commercial and validated by manufacturers, and most include controls and calibration curves. It is less clear whether a single sample and IFN subtype, such as serum IFN-alpha, is the most clinically relevant to a patient.

Concurrent validity: Concurrent validity was provided in 12/57 reports IFN-α and 4/9 for IFN-β against other assays such as expression of individual ISG,^18 19^ ISG expression scores,^20^ soluble IP-10 levels,^21^ flow cytometric measurement of SIGLEC1,^22^ bioassays,^23^ virus inhibition assays^24^ or reporter cell assays. Outcomes of these comparisons were highly variable. Some showed no clear association with IFN-α levels (ie, for IP-10, expression of individual genes),^20 21 25 26^ weak associations (IFN inducible gene expression, bioassay),^20 21^ or strong association (ie, between RIA and virus inhibition assays or SIGLEC-1 by flow cytometry or IFN-α and expression of individual ISGs).^19 22^

### SiMoA (single molecule assay)

Eight studies were identified.

Content: A proprietary technology for ultrasensitive digital protein detection with a commercial assay for IFN-α (and some IFN-induced proteins).

Criterion validity: This was shown in six of eight studies. The study by Rodero *et al* showed that SiMoA could detect all subtypes of IFN-α, and excluded crossreactivity testing antibodies against recombinant IFN-β, IFNλ−1, IFNλ−2, IFN-ω and IFN-γ. This paper also described criterion validity showing that addition of anti-IFN-α antibody depleted the signal. Reproducibility was also confirmed. Matched plasma and serum samples tested for IFN-α showed strong correlation indicating a negligible influence of blood processing on IFN-α concentration and the ability to use either sample for retrospective patient screening. The potential implication of missing non-circulating sources was emphasised since intracellular IFN-I was detected in samples from patients with gain of function mutations in Stimulator of IFN Genes (STING) but not SLE despite high expression of ISGs in both. The IFN-α concentrations that are detected by SiMoA are often below the levels required to induce a cellular response in vitro.

Concurrent validity: This was reported in five of eight studies. SiMoA IFN-α measurements were highly correlated with a 6 ISG qPCR score as well as Nanostring and soluble Siglec-1.^3 27 28^ IFN-α protein by SiMoA correlated with cytopathic effect using Madin–Darby bovine kidney cells challenged with vesicular stomatitis virus.^3^ In some SLE cases, SiMoA detected IFN-α while a bioassay was negative, so the biological significance of the low IFN-α concentrations detected by SiMoA may require further confirmation.

### Immunoassays: IFN-inducible proteins

Forty-two studies were identified.

Content: Immunoassays for soluble proteins encoded by ISGs in biosamples including ELISA, DELFIA, multiplex assays and radioimmunoassays.

**Figure 4:**
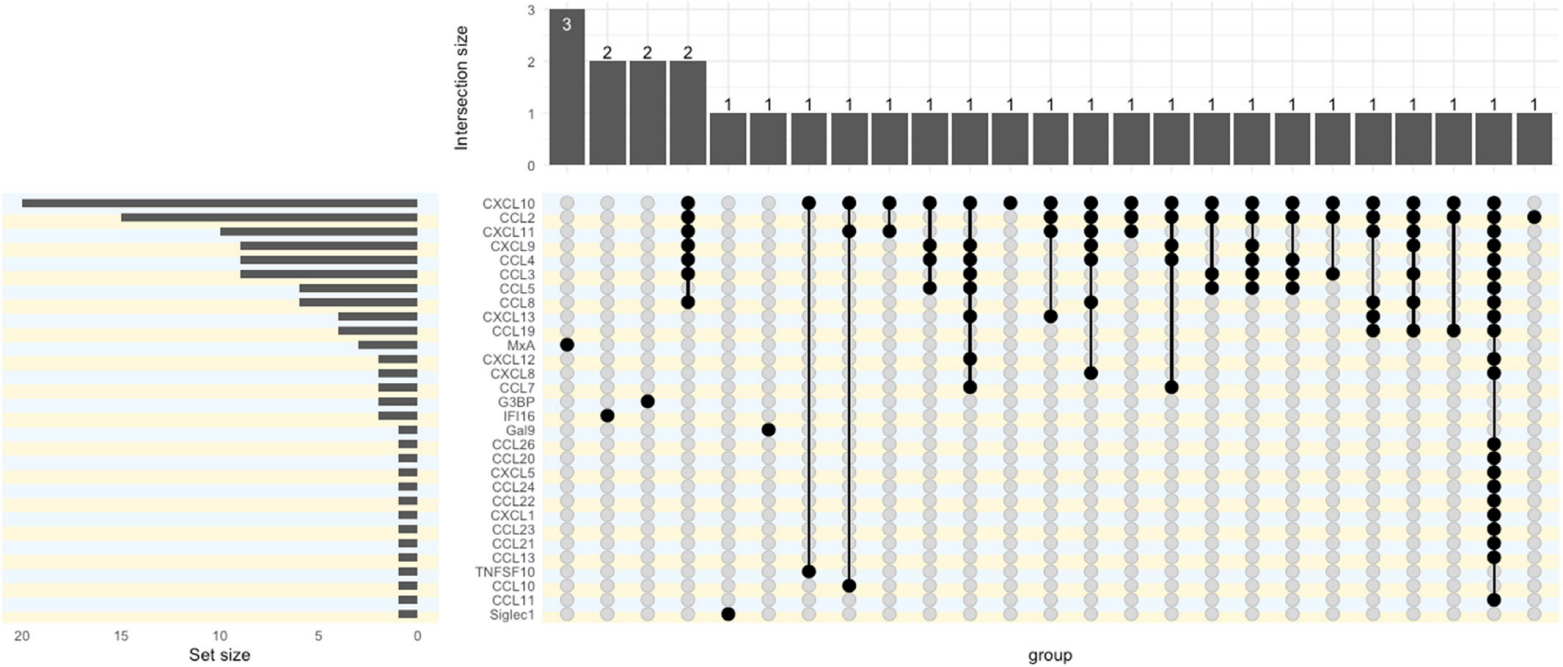
UpSet plot for constituents of immunoassays. Dots and bars indicate what combinations of proteins were measured in reports of IFN immunoassays. The left-hand chart shows the number of reports for each protein. The upper chart shows the number of reports for each combination.

Criterion validity: These assays do not evaluate IFN-I directly and production of some of the IFN-inducible proteins reported is known to be also induced by IFN-II or other inflammatory mediators. This is likely a particular problem if a single ‘IFN-inducible’ protein is reported. Of 42, 21 studies measuring chemokines cited papers showing they are IFN-I-induced. Only 3/42 studies provided experimental evidence. Bauer *et al*^29^ selected IFN chemokines whose transcripts were induced by IFN-α in a microarray study but did not check whether other IFNs or inflammatory mediators also induced these proteins. Thanarajasingam *et al*^30^ evaluated stimulation of whole blood including IFN-α, oligonucleotides with cytosine‐guanine repeats, Resiquimod (R848), LPS, IFN‐α+LPS and null (no stimulant) then measured cytokines/chemokines and IFN-α. Most of the other studies cite the evidence from Bauer *et al*.^29^ The IFN-inducible proteins included in assays are summarised in figure 4.

Concurrent validity: Of 42, 16 studies demonstrated concurrent validity against another test for IFN-I. 11/16 used an ISG expression assay.^29–38^ In two studies concurrent validity was tested between the IFN-inducible protein and two other techniques (flow cytometry for SIGLEC-1^22 39^ as well as IFN-α protein^21 26^; and gene expression as well as a reporter cell assay).^30 40^

### Flow cytometry

Twelve studies reported using flow cytometry to analyse IFN-I-inducible markers.

Content: Evaluation of IFN-inducible proteins, mostly on the cell surface, using flow cytometry. It allows cell-specific measurement that avoids artefacts due to changes in the cellular composition of the sample. The most common target was SIGLEC-1. Other markers reported were CD64 (FCGR1), MxA, IFITM1, PRKRA. Only monocytes can be analysed using SIGLEC-1 (CD169), so monocyte data represent the bulk of this literature.

Criterion validity: Of 12, 4 studies reported in vitro experiments showing stimulation of cells (PBMCs cells subsets or cell lines) with IFN-I and induction of the flow cytometry markers. Of 12, 9 studies cited evidence from the literature that the genes encoding the flow cytometry marker are IFN-inducible, or prior papers that report the marker as an IFN assay. These studies did not report whether other subtypes of IFN or other inflammatory mediators induced the flow cytometry markers.

Concurrent validity: Of 12, 5 studies demonstrated correlation with another IFN assay such as the same protein in serum by ELISA, the expression of the protein’s transcript by qPCR, against IFN-α protein measured by ELISA, or against IFN scores derived from qPCR assays.^22 35 39 41–43^ Some comparator assays compared ELISA or IFN Score results from unsorted blood with a specific cell population analysed by flow cytometry.

### RNA microarrays

Microarrays were reported in 70 studies in total, with several differences in the methods section.

Content: A gene expression assay using probes that provide broad coverage of the transcriptome.

Of the 70 studies, 3 main methods of data analysis were used: (1) reporting an ‘IFN signature’, referring to the presence or absence of a cluster of ISGs, usually readily visible on a heat map; (2) an ‘IFN score’, usually referring to a continuous variable calculated from a predefined set of ISGs or (3) modules of ISGs, which allows the clustering of ISGs into two or more groups, and then a signature or score to be provided for each module or differences across modules are screened. The terms for these methods have not always been consistently used.

Of 70, 36 studies reported IFN signatures, 23/70 as IFN scores and 10/70 studies presented results as IFN modules. The most popular platform used was Affymetrix (39/70), followed by Illumina (15/70), Stanford microarrays (4/70), Agilent (3/70) or custom-made arrays. Microarrays were performed mainly using RNA from whole blood (41/70). Blood was collected using PAXgene (36/70) or Tempus tubes (5/70). Others used PBMCs or various combinations of isolated cell subsets.

Criterion validity: Of 70, 16 studies presented stimulation experiments showing induction of ISGs by IFNs-I or used public datasets to derive ISG sets. A few studies also reported ISG downregulation or IFN signature neutralisation after experiments or administration of IFN-targeted medications. The modular analysis provided data suggesting that modules may represent the relative abundance of type I versus type II IFNs. As for all gene expression assays described, a potential limitation when analysing unsorted cells is artefactual change due to changes of the cellular composition of the sample. Indeed, ISG expression differs between cell subsets and patients with RMDs often have cytopenias or expansion of cell subsets secondary to autoimmunity or immunosuppressive therapy.

Concurrent validity: Provided in 33/70 reports. Microarray results were validated against qPCR scores, individual ISGs expression, serum IFN-α, -β and IFN-inducible chemokines and a methylation array.

### RNA-sequencing

Nine studies reported RNA-sequencing (RNASeq).

Content: Sequencing of the whole transcriptome providing qualitative and quantitative data on any RNA type.

Studies reported differentially expressed genes including ISGs in whole blood (3/9), isolated CD19+B cells (3/9), monocytes CD14+, pDCs, neutrophils (1/9 each subset). RNASeq was performed on Illumina.

Criterion validity: As for other gene expression methods. Stimulation with IFN-I was shown in one out of nine study, in another ISGs were derived from the Interferome database. ISG were not reported in subsets as for micro-arrays and qPCR studies.

Concurrent validity: provided in three ouf of nine reports, against chemokine score in serum,^44^ methylation profile of selected ISGs^45^ and qPCR.^46^

### Nanostring

Five studies reported gene expression using Nanostring.

Content: A proprietary probe-based gene expression usually analysing 800 transcripts in manufacturer designed or customisable panels.

Nanostring was reported in in five out of nine studies to evaluate expression of ISGs in whole blood, PBMCs and CD19+B cells.

Criterion validity: as for other gene expression assays. Commercial predefined panels were provided by the manufacturer.

Concurrent validity: Provided in four out of nine studies where results were compared with serum protein IFN-α or SIGLEC1, IFN-I scores by qPCR or RNA-seq.^28 39 46 47^

### IFN-I scores by qPCR

qPCR was reported in 122 studies with differences in the methods section.

Content: All qPCR studies report a set of real-time PCR techniques based on commercial or custom probes and primers for quantification of predefined ISG transcripts. Of the 122 studies, 5 main methods of data analysis were used: (1) 4/122 measured qPCR for genes encoding IFN proteins; (2) 30/122 reported expression of individual ISGs; (3) 82/122 studies reported an IFN Score—that is, expression of a set of ISGs as a continuous value; (4) 4/122 studies reported IFN-inducible chemokines as scores or individual genes; (5) 2/122 reported clusters of ISGs as a categorical signature.

**Figure 5:**
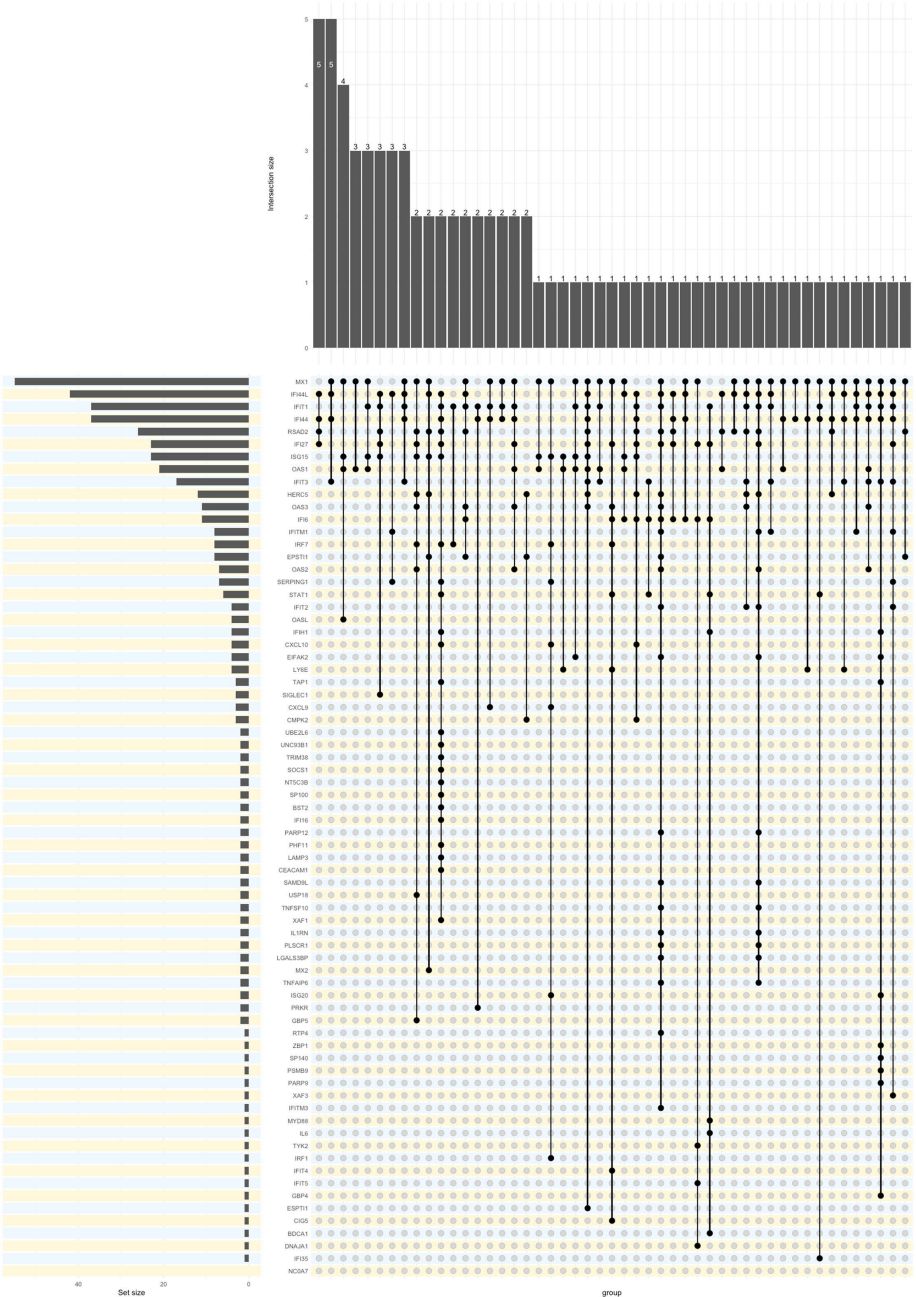
UpSet plot for constituents of gene expression assays. Dots and bars indicate what combinations of genes were measured in reports of IFN gene expression assays. The left-hand chart shows the number of reports for each gene. The upper chart shows the number of reports for each combination.

Criterion validity: as for other gene expression assays. A total of 14/122 studies contained direct evidence based on experimental stimulation with appropriate positive and negative controls. The ISGs and reference genes chosen were highly variable between studies, and the rationale for these choices was not always given. A list of transcripts in scores used in RMDs is shown in figure 5. Sources and preanalytical features also differ across studies.

Some papers derived and used more than one score, related to the modular data from microarray studies or from unsupervised approaches (factor analysis or principal component analysis). Many studies reported correlations among ISGs, providing additional criterion validity. While many papers suggest these reflect balance of IFN-I and IFN-II, this was not demonstrated in any paper.^46 48 48 49 49 50 50–54^ However, when multiple scores were analysed, particular sets of ISGs, such as IFN-Score-B, show a stronger correlation with clinical endpoints than others in certain conditions. The metric properties of the scores, dynamic ranges and calculations to derive them may be other reasons that some sets of ISGs provide better clinical correlations, which also applies to other gene expression assays.

Concurrent validity: Provided for 43/122 studies, mainly by comparing new gene expression scores against previously published scores that use different ISGs. Several studies validate scores against levels of IFN-α protein measured by ELISA, SiMoA, etc); IFN-stimulated proteins (SIGLEC1, GAL9, G3BP); chemokines/chemokine scores; or expression of individual ISGs (MxA).

### DNA Methylation arrays

Fourteen studies analysed an aspect of the IFN pathway using DNA methylation arrays.

Content: Analysis of methylation of genomic DNA to identify changes in actively transcribed genes

The material analysed included isolated cell subsets (most frequently CD4+T cells, but also CD8+T cells, CD19+B cells or neutrophils) as well as whole blood and PBMCs. The Illumina Human Methylation 450 BeadChip (HM450) was the most commonly method used for high-throughput human methylome analyses in 13/14 studies. Other studies used a newer Illumina assay based on the EPIC chip (Illumina Infinium MethylationEPIC BeadChip). Differential methylation was reported for ISGs as well as *IFNA* gene across all tested blood cell types and samples.

Criterion validity: These assays do not directly measure the transcription of IFNs or ISGs, nor their protein products so are only an indirect indication of activation of IFN-related pathways based on prior knowledge. The studies retrieved did not add any direct evidence to support their specificity.

Concurrent validity: Of 14, 10 reports presented concurrent validity. In three cases, results were validated against bisulphite sequencing, pyroseqencing and others looked at impact of hypomethylation on gene expression by qPCR (comparing to selected representative individual genes) or microarray results (IFN score) or RNA-seq. One study validated methylation results against IFN-α and -β protein levels in serum. Of 14, 2 studies validated their results against publicly available methylation datasets.

### Reporter cell assays

Forty studies reported data from reporter cell assays.

Content: Quantitative analysis of the ability of IFNs in biosamples (ie, serum) to upregulate ISGs in a reporter cell line.

Various combinations of cells, biosamples and methods of readout have been published. Most studies analysed serum, with plasma in a smaller number. Samples were used to stimulate a reporter cell.

Two main groups of reports were identified. A first group of 31 studies used reporter cell assays with a qPCR readout. Most of these used a WISH cell line (human amnion) combined with patient serum (22 studies); 4 studies used HeLa cells; others used PBMCs from healthy controls, THP1 cells or HUVEC. The output readout was measured by qPCR using combination of 1–6 ISGs. The ISGs and reference genes chosen were highly variable between studies, and the rationale for these choices was not given. Results were usually presented as individual gene expression or IFN scores. The ISGs and reference genes chosen were highly variable between studies, and the rationale for these choices was not given. Results were usually presented as individual gene expression or IFN scores.

A second group of nine studies used measurement of output by luminescence or spectrophotometry. In this group, three studies used A549 cells (human lung carcinoma); two used novel HEK-Blue IFN-α/β and/or donor PBMCs; one used Hil3 (hepatoma cell line); one used U937 cells stably expressing an Mx1 promoter; one used U937-Mx1-Luc (containing luciferase reporter construct) and one used Fibroblast cells (FL). The results were presented in various formats such as: ‘Activity’ (titres, dilutions or arbitrary units) or concentration of IFN-α, etc.

Criterion validity: From both subcategories of reporter cell assays 14/29 and 2/9 gave experimental evidence on measuring IFN-I by using positive controls, inhibition with anti-IFN-I antibodies, stimulation with IFN-I or describing using a standard curve.

Concurrent validity: 8/29 and 5/9 studies, respectively, from these two groups presented concurrent validity. In most cases this was compared with IFN scores by qPCR measured in PBMCs or whole blood as well as against IFN-α protein levels in serum or plasma

### Cytopathic effect assay

Eleven studies reported cytopathic effect assays.

Content: Measures the capability of IFN-I in a biosample to suppress the cytopathic effect of viral replication on a target cell in vitro. Various combinations of cells and viruses have been reported (discussed in online supplemental text 6). The most common combination is the human lung carcinoma cell line A549 challenged with encephalomyocarditis virus (EMCV).

Criterion validity: All studies used IFN-I standards and further three additionally used neutralisation of IFN with anti-IFN-α antibodies.

Concurrent validity: Three reports provided evidence of concurrent validity against SiMoA and RIA IFN-α protein measurement.

### Plaque reduction assay

Content: Evaluation of the ability of IFN-I in a biosample to prevent cell killing by a lytic virus in vitro. This assay was used in n=7 RMD studies. Many different combinations of cells, patient sample types and methods of readout have been published. The cell line used most frequently was WISH in combination with serum and vesicular stomatitis virus (VSV) (n=5). Two other studies used fibroblastic cell lines (human foreskin fibroblasts (HFF) ormammary stromal fibroblastic (MSF) cell lines) with VSV. All these studies were published in 1970s and 1980s. The last study identified, from 2011, reported using VERO cell line in combination with serum and EMCV.

Criterion validity: All eight studies reported using IFN standards (recombinant IFN-α mainly, IFN-β and IFN-γ in some cases) as positive controls, as well including negative controls. A neutralisation assay with anti-IFN antibodies was also performed in three cases.

Concurrent validity: nNne of studies showed results of concurrent validity

### IHC

A single study reported the results of whole blood stained for MxA as semi-quantitative intensity of staining. This assay shares similar considerations to other assays for single IFN-stimulated proteins.

### Feasibility

Key considerations in feasibility provided by the task force members after reviewing manuscripts is provided in online supplemental table 1.

### Definitions of terms

The terminology used to describe aspects of IFN-I pathway activation was not consistent in the literature eligible for this SLR. This includes the distinction between signatures and scores, abbreviations for IFN subtypes and even the use of the term ‘interferonopathy’ (used to indicate either monogenic diseases, but also to refer to any polygenic RMDs in which increased IFN pathway activation was observed, especially SLE). Having identified key areas with inconsistent nomenclature and reporting, consensus terminology was agreed through an interactive compromise process with reference to the existing evidence. Recommended terms relating to IFN-I reporting in basic and clinical research is presented in table 2.

**Table 2:** Consensus terminology

Term	Abbreviation	Definition
Interferon	IFN	Proteins with antiviral activity; IFNs are mediators of an antiviral response. They belong to the type I, type II and type III IFN families.
Type I interferon	IFN-I	The IFNs alpha, beta, omega, kappa, epsilon, secreted by any nucleated cell, and binding to the IFNAR, which is expressed on any nucleated cell.
Type II interferon	IFN-II	IFN gamma, mostly secreted by T cells, binding to the IFNGR, which is expressed on most leucocytes.
Type III interferon	IFN-III	IFN lambda, which are structurally more similar to IL-10 but share downstream signalling and gene expression with IFN-I.
Interferon-stimulated genes	ISGs	Genes whose expression is known to be upregulated by any kind of IFN. Individual ISGs may not exclusively represent type I IFN pathway activation.
Type I Interferon pathway		Type I IFN pathway is a dynamic, biological system that includes the secretion of type I IFN protein, binding to the IFNAR, initiation of JAK/STAT signalling pathways, expression of IFN-stimulated genes and the expression of IFN-stimulated proteins.
Type I Interferon pathway activation		Any evidence for changes in function or levels of the components of the Type I IFN pathway.
Type I interferon pathway assay		An assay measuring one or more components of the type I IFN pathway at a molecular or functional level.
Interferon stimulated gene expression signature		A qualitative description of coordinated expression of a set of ISGs that is indicative of type I IFN pathway activation.
Interferon stimulated gene expression score		A quantitative variable derived from expression of a defined set of ISGs that is indicative of type I IFN pathway activation.
Interferon stimulated protein score		A variable derived from expression of a defined set of soluble biomarkers known to be upregulated by IFN, although not specific for type I IFN.
Interferonopathy		Mendelian diseases in which there is constitutive type I IFN pathway activation with a causal role in pathology. The clinical picture may resemble RMDs. However, most diseases with IFN pathway activation are polygenic disorders and not mendelian Interferonopathies.

ISG, IFN-stimulated genes; RMDs, rheumatic musculoskeletal diseases.

## Discussion

This SLR is the first summary of the entirety of the literature of IFN-I assays in the field of rheumatology. We identified a substantial body of literature supporting the value of these assays, but simultaneously several inconsistencies in the literature that are a barrier for the progression of these biomarkers into clinical practice. These findings provide a knowledge framework to guide assay selection and will inform the development of EULAR points to consider for the measurement, reporting and application of IFN-I pathway activation assays in clinical and research practice.

The assays described all measure different aspects of the complex IFN pathway. However, none of them can assess all relevant elements of the IFN-I activity in a human patient, and many of them are not specific for IFN-I. Hence, no single assay can evaluate the entirely of the IFN-I pathway activation. The most appropriate assay choice is contextual, depending on the specific research or clinical question as well as aspects of feasibility and reliability.

**Figure 6:**
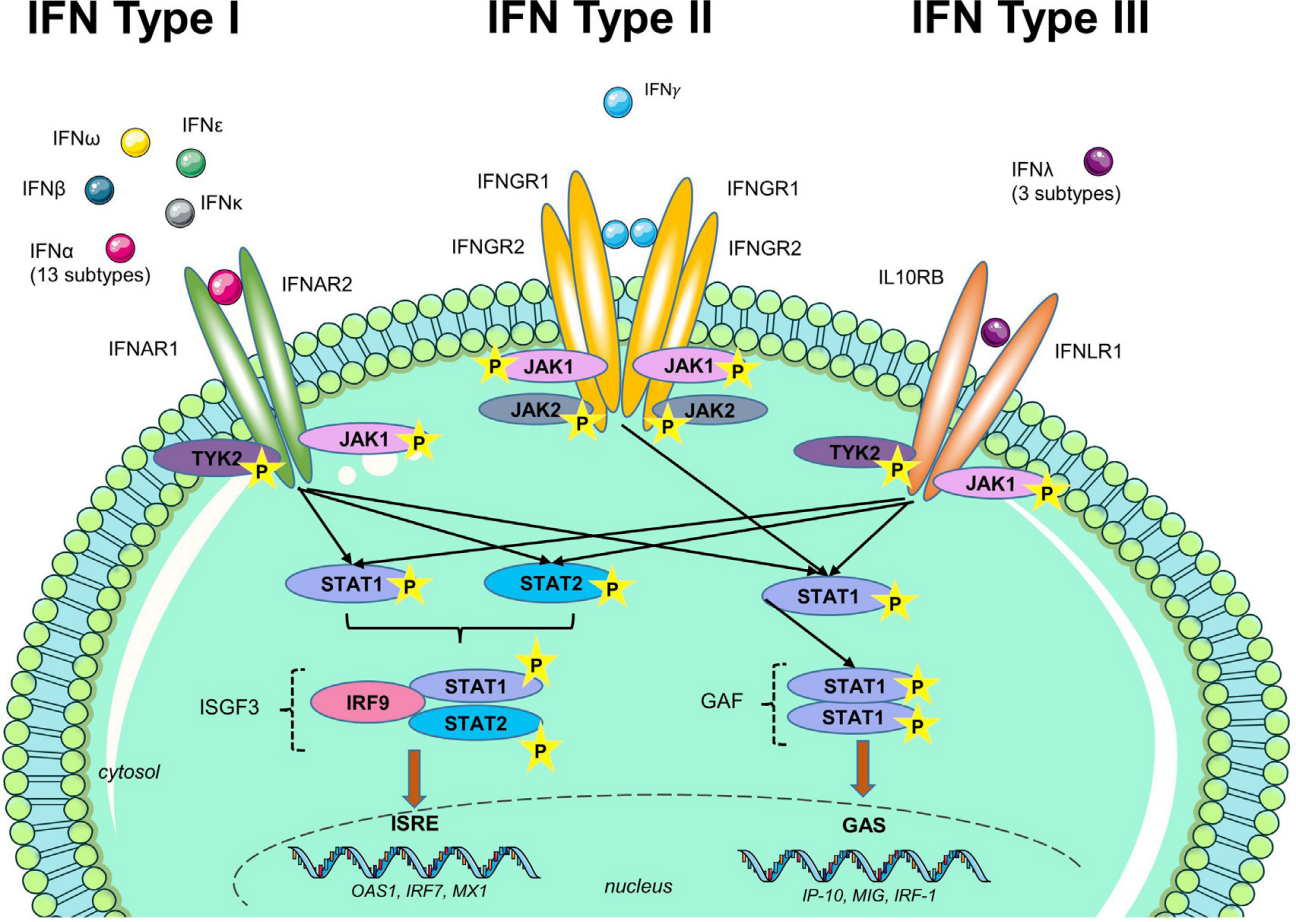
Overlap between types of IFNs. Although there are distinct receptors for type 1, 2 and 3 IFNs, there is substantial overlap in signalling and response elements. Assays measuring segments of the pathway downstream from the IFN receptor may not be specific for one subtype of IFN and some may preferentially reflect type II or III IFNs.

The complexity of this pathway presents an intrinsic problem in construct validity, that is, evidence that putative IFN-I assays measure IFN-I pathway activation specifically. Given that no assay can measure the entirety of IFN-I pathway activation, there is no ‘Gold Standard’ against which to evaluate new assays. Although SiMoA has increased the ability to measure IFN-I proteins themselves in the circulation more easily, this is only one component of the pathway. Further, non-circulating sources of IFN-I may be more important in many contexts. Molecular events downstream of the IFNAR may be more relevant to immunopathogenesis and thus, clinical applications. Meanwhile, assays for downstream molecules, such as assessment of ISG expression of IFN-inducible proteins, may be responsive to other IFNs or cytokines (Figure 6). Many of the papers reviewed presented concurrent validity (ie, correlation with another putative IFN-I assay) as evidence to support that they are IFN-I assays. This is weak evidence. The effects of IFN-I on the immune system and other aspects of its biology are so diverse that many parameters –ranging from acute-phase markers to symptom scores—may be found to correlate with IFN-I pathway activation. For this reason, methods for construct validity are likely to be measures of criterion validity, such as in vitro stimulation of cells with IFN-I as well as negative and positive controls before evaluating gene expression; or inhibition of reporter cell assays with anti-IFN-I antibodies. These methods do not represent autoimmunity in the complete organism. Regardless of these issues, representation of the complete IFN-I pathway may not be necessary for a clinically applicable biomarker.

An additional aspect of analytical validity that varied between reports was the analysis and reporting of results. Many papers reporting gene expression assays presented results as high or low, or positive or negative; and several cut-off criteria and score calculation practices were proposed. Although ISG expression is often bimodally distributed, this dichotomisation remains a dubious concept, since cytokine levels and cellular responses are naturally continuous. The bimodal distribution of results may differ in non-SLE RMDs, or in specific SLE populations. One study demonstrated that evaluation of IFN scores as continuous variables provided clinically relevant information in addition to high/low status (ie, very high expression has a different clinical significance to moderately high^50^).

Immunoassays are often used to assess directly IFN levels in biosamples from patients with RMDs. However, sensitivity of these methods has been questioned in the past since IFN-I may be below detection^55^ when assayed using ELISA. This method is also influenced by low reproducibility and shows rather variable correlation with bioassays^55 56^ possibly because of other subtypes of IFN or because the assay can detect a similar epitope on a non-IFN-α protein, a stable but biologically inactive IFN-α protein degradation product^56^ or the presence of other IFN subtypes. Of note, these limitations must be regarded as antibody specificity-related rather than to the assay itself. While the SiMoA technology can measure proteins with an increased sensitivity a limitation is the lack of a commercially available kit to detect all IFN-I subtypes. And levels of IFN in serum may not be the only relevant IFN protein influencing IFN pathway activation. Clinical validation to demonstrate any superior properties as a biomarker is still required.

Three main types of assay to evaluate downstream cellular responses to IFN-I had the most substantial body of literature; immunoassays for soluble IFN-stimulated proteins, assays for IFN-stimulated genes and flow cytometric assays for IFN-stimulated cell surface proteins. For all of these, confirmation that they pertain specifically to IFN-I is critical and not always provided. However, these assays generally appeared feasible in routine practice. One additional flow cytometry marker was published after completion of the SLR, which is evaluable on any cell subset.^4^

Functional assays or bioassays are among the oldest in the literature with more modern adaptations in later papers. These assays have obvious interest in terms of biological significance, but most appeared to have poor feasibility for routine clinical practice.

This SLR has some limitations. Due to the heterogeneity in the methods of reporting, it was not possible to perform meta-analysis. The presentation format of the results representing the same assay was so variable that did not allow for comparison of data between studies. Moreover, assays were evaluated on the basis of their methodologies and technical aspects, whereas their clinical associations were the focus of other SLR (reference to SLR2). Although some papers reported more than one assay or method (2–4), these did not necessarily provide concordant results. Some studies described multiple assays as independent assays of IFN-I pathway activation. Reports also varied by sample types and study designs, and assay usage differed across RMDs. Furthermore, there is a lack of standardised instruments for comparative analyses and specific technical aspects, such as feasibility. For these reasons, we relied on expert commentary in this SLR.

This SLR is the first step in a programme designed to facilitate translation of IFN-I assays in clinical research and practice. Together with an analysis of their associations with clinical outcomes, these will inform the EULAR points to consider for the measurement, reporting and application of IFN-I pathway activation assays in clinical and research practice. We believe this programme will ultimately lead harmonisation and to widespread adoption of these promising assays to improve patient care. Although genetic studies were not included in this review, we consider that our task force will also facilitate progress in the characterisation of genetic variants. Moreover, due to the broad involvement of IFN in human disease, our analysis may be also applicable beyond the field of rheumatology.

## Data Availability

All data relevant to the study are included in the article or uploaded as supplementary information.
