# Supplementary material for: Type I interferon pathway assays in studies of rheumatic and musculoskeletal diseases: a systematic literature review informing EULAR points to consider
Source: RMD Open. 2023 Mar 2;9(1):e002876. doi: 10.1136/rmdopen-2022-002876 (PMC9990675; doi:10.1136/rmdopen-2022-002876)
Supplement: Supplementary data [file rmdopen-2022-002876supp001.pdf]

SUPPLEMENTARY MATERIAL

Supplementary Table 1: Feasibility of IFN-I assay subtypes
Supplementary Text 1: Research questions (PICO framework)
Supplementary Text 2: Search Strategy for Ovid MEDLINE
Supplementary Text 3: Search strategy for EMBASE
Supplementary Text 4: Search strategy for Web of Science
Supplementary Text 6: Inclusion and exclusion criteria
Supplementary Text 6: Detailed description of assay methodology

Supplementary Table 1: Feasibility of IFN-I assay subtypes

Method and element of IFN pathway evaluated	Material	Cost	Equipment	Technical difficulty	Issues affecting reliability
Immunoassay for IFN-α, IFN-β, or IFN-inducible soluble proteins	Serum or plasma are easily to obtain and processed and can be stored in -80C until analyzed.	Relatively low depending on the numbers of proteins measured and whether ELISA kits or multiplexing platform used	ELISA equipment is widely available. For multiplex panels more specialised equipment might be required	ELISA and multiplex systems are usually commercial kits widely used in research and clinical laboratories which are optimized with simple protocols. RIA requires special precautions and licensing, since radioactive substances are used.	Commercial assays are validated by manufacturers for intra and inter-assay variability. ELISA is sensitive to minimal artefacts in pipetting or preparation, which can be mitigated by duplicate analysis.
SiMoA for IFN-α, IFN-β, or IFN-inducible soluble proteins	Serum or plasma are easily to obtain and processed and can be stored in -80C until analyzed. Samples must be processed and frozen within 4 hours	Expensive compared to other assays. Important cost for antibodies kits and equipment (both acquisition and maintenance of the SiMoA machine). Staff time dedicated to the assay.	Specialised equipment required. Limited by antibodies commercially available (so far, only anti-IFN-α2 antibody available, pan-IFN-α antibody not commercially available, lack of sensitivity for IFN-β)	The technique relies on commercially available antibodies (i.e. pan-IFN-α antibody not commercially available at the time of this SLR). SiMoA requires optimization and definition of control values for each equipment and antibody used.	Relatively young technology, bringing some issues: in particular, no standardization for normal range values.<br><br>Results also depend on the equipment used and the antibody used, so that reproducibility and comparison of results and values for healthy individuals between centres is challenging.<br><br>Autoantibodies against a subtype of IFN-I may also affect the results.<br><br>Lack of sensitivity for IFN-β.
Flow cytometry for IFN-inducible	Whole blood or isolated PBMCs, which must be	Medium cost for reagents depending on numbers of	Flow cytometers are required although available in research	Flow cytometry requires training to optimize panels	Flow cytometry is difficult to standardize within and between centres. Key issues

**Method and element of IFN pathway evaluated**	**Material**	**Cost**	**Equipment**	**Technical difficulty**	**Issues affecting reliability**
proteins on cell surface	analysed or processed and frozen within hours. Separation of PBMCs is only available in certain centres.	antibody markers chosen. Staff time is more expensive than assays that analyse in batches.	institutions and clinical immunology labs.	and capture data, although this is widely available.	are transportation, time to anlaysis, sample preparation, instrument calibration, subjective elements in analysis. Newer flow cytometry instruments and reagents that help address these problems.
IFN-stimulated genes detected by microarrays	RNA may be obtained from various patient samples. Preserving RNA integrity requires timely processing. However the use of tubes with RNA stabilizing agents allows easy collection and transport from any site when using whole blood.	Expensive compared with other gene expression assays. Cost is also dependent on microarrays choice (self-produced or customized vs commercially available)	RNA isolation requires a nuclease-free environment and appropriate extraction kits with DNA removal.<br>Dedicated equipment needed for scanning and analyze microarray chips.	Microarray analysis follows established protocols, usually with commercial kits and equipment. Error-prone steps may require replication. There are examples of translation of micro-array signatures into clinical practice in various specialties..	Quality of sample preservation affects gene expression assays. Background correction and normalization must be monitored to ensure comparability across genes with different dynamic ranges and expression rates.
qPCR for IFN-stimulated genes	RNA may be obtained from various patient samples. Preserving RNA integrity requires timely processing and expertise. However the use of tubes with RNA stabilizing	Relatively cheap depending on number of transcripts selected for analysis and batching.	RNA isolation requires a nuclease fee environment and appropriate extraction kits with DNA removal.<br>Thermocycler, qPCR analyser and software are required which are widely available in	qPCR is widely used and usually follows commercial protocols, although in-house assays require additional skill to optimize.	Quality of sample preservation affects gene expression assays.<br>Commercial assays are validated by manufacturers for intra- and inter-assay variability. qPCR is sensitive to minimal artefacts in pipetting or preparation, which

Method and element of IFN pathway evaluated	Material	Cost	Equipment	Technical difficulty	Issues affecting reliability
	agents allows easy collection and transport from any site when using whole blood.		research and routine clinical laboratories.		can be mitigated by duplicate analysis.<br>Data analysis and reporting practices are not uniform and may introduce artifacts.
RNASeq for IFN-stimulated genes	RNA may be obtained from various patient samples. Preserving RNA integrity requires timely processing. However the use of tubes with RNA stabilizing agents allows easy collection and transport from any site when using whole blood.	For analysis focused on ISG expression RNASeq is expensive and complex and provides similar information compared to qPCR. However, there is added value from the information about non ISG expression and other species provided.	RNA isolation requires a nuclease-free environment and appropriate extraction kits with DNA removal.<br>Sequencing equipment needed (usually outsourced)	Sample preparation, library preparation, acquisition and data analysis are technically complex requiring specialist training and considerable time.	Quality of sample preservation affects gene expression assays.
Nanostring for IFN-stimulated genes	RNA may be obtained from various patient samples. NanoString method does not require polymerase activity, therefore is suitable for less-than-optimal materials/samples such as: lysates, plasma, or FFPE	Nanostring assays are more expensive than qPCR but automated analysis reduces cost, and it is cheaper and easier to analyse compared to RNAseq or micro-arrays.	RNA extraction in nuclease-free environment. Prep Station and Digital Analyzer (NanoString nCounter Analysis System) are required, which are currently only available in a limited number of centres.	Specialist training is required but a standardised protocol and preparation station provided from the manufacturer that reduces sample handling and introduction of errors. Pre-prepared validated panels reduce laboratory handling and possibility to introduce errors to minimum. Unlike qPCR, RNASeq and micro-	The NanoString technology is a robust, sensitive, reproducible. The lack of library preparation and pre-amplification reduces risk of bias compared to some other gene expression methods.

Method and element of IFN pathway evaluated	Material	Cost	Equipment	Technical difficulty	Issues affecting reliability
	samples, and damaged or old samples.			arrays, no library preparation, additional enzymes or pre-amplification steps, or other processing are not required.	
DNA methylation for IFN-stimulated genes	Most studies used easily accessible material such as whole blood, cell subsets or tissue. DNA has to be extracted using standardised laboratory methods such as column method. Method requires low input of starting material (500 ng for HK450 and 250ng for EPIC) and DNA has to be bisulfite-converted.	Relatively expensive. The minimum number of samples per kit is 24, and the chip for hybridization accommodates 12 samples. Hence the costs are relatively high compared to other assays. Analysis is frequenctly outsourced.	DNA extraction in nuclease-free environment.<br>Sequencing facilities private centers/companies offer services (frequently outsourced).	Usually performed with commercial kits, following established protocols, with manufacturer-provided chips and analysis pipelines.	Background literature on DNA methylation reports documented *patterns* of uneven reliability in the repeated measurement of DNA methylation. Hence this method is usually used at a discovery stage and more robust methods of analysis methylation should be used for validation and application to detect biomarkers.
Reporter cell assays to evaluate for IFN-α, IFN-β in a patients sample using gene expression or colorimetric changes as a readout	Serum or plasma are easily to obtain and processed and can be stored in -80C until analyzed.	Relatively cheap, particularly when large numbers of samples are analysed in parallel.	Tissue culture facilities needed.	Requires tissue culture skills. Complex with multiple stages where errors can be introduced. Careful optimisation of the assay is required.	Significant issues due to lack of standardisation and complexity. Therefore might not be applicable to clinical settings.

**Method and element of IFN pathway evaluated**	**Material**	**Cost**	**Equipment**	**Technical difficulty**	**Issues affecting reliability**
Cytopathic effect assay to evaluate for IFN-α, IFN-β in a patients sample using cytopathic changes as a readout	Serum or plasma are easily to obtain and processed and can be stored in -80C until analyzed. A cell line that is sensitive to IFNs of interest is also needed.	Time-consuming and labour intensive compared to some of the other IFN-I assays in the literature.<br>Due to facilities and staff time required, costs are relatively high. Routinely available in some centres.	Biosafety containment such as biohazard cabinets to safely handle viral infections, as well as and sterile tissue culture conditions to avoid coinfections are required.	Requires skills in sterile tissue culture techniques.	Cytopathic effect assays are affected by the subjective nature of determining endpoints, that prohibits calculation of exact titers.<br>Some commercial assays are available that are more cost-effective and time-efficient assay suitable high throughput screening of overall antiviral activity.
Plaque reduction assay to evaluate for IFN-α, IFN-β in a patients sample using plaque size as a readout	Serum or plasma are easily to obtain and processed and can be stored in -80C until analyzed. Also requires a selected cell line to grow in monolayer, and virus able to produce plaques on confluent host cells.	Time-consuming and labour intensive compared to some of the other IFN-I assays in the literature.<br>Due to facilities and staff time required, costs are relatively high.	Biosafety containment such as biohazard cabinets to safely handle viral infections, as well as and sterile tissue culture conditions to avoid coinfections are required.	Requires skills in sterile tissue culture techniques. Optimisation of the protocol for a particular viral culture is critical.	Several issues may affect standardization including analysis method and control choice.
IHC to evaluate IFN-stimulated proteins in whole blood	Fixed smear of whole blood	Not expensive, but dependent on antibody choice.	Standard IHC dedicated equipment is commonly available.	Skills in IHC are widespread in research and clinical laboratories	Numerous issues may affect reliability including the quality of the blood smear, the optimization of the staining, and subjective elements to evaluation.

Supplementary Text 1: Research questions (PICO framework)

Research questions	***Population***	***Intervention (Exposure)***	***Comparison***	***Outcome***
**What is the evidence that interferon measurement is useful in the diagnosis of RMDs?**				
	People presenting with any RMD<br><br>Control population	IFN pathway assay measurement	RMD vs control population	Association between measurement of IFN pathway activation and RMD diagnosis
**What is the evidence that interferon measurement reflects disease activity in RMDs?**				
	People presenting with any RMD	IFN pathway assay measurement	Disease activity measurement	Association between measurement of IFN pathway activation and disease activity (difference between groups, association, correlation)
**What is the evidence that interferon measurement is useful for the prognosis (natural history) of clinical status in RMDs?**				
	People presenting with any RMD, 'at-risk' of RMDs or in preclinical stages	IFN pathway assay measurement	Disease exacerbation / flare vs no disease exacerbation / no flare<br><br>Progression to clinical RMD (fulfillment of classification criteria)<br><br>Severity of the clinical course (occurrence of comorbidity or organ	Association between measurement of IFN pathway activation and disease exacerbation, progression to clinical RMD or severity

			involvement or damage)	
**What is the evidence that interferon measurement is useful for the prognosis (response to treatment) in RMDs?**				
	People presenting with any RMD starting a new therapy	IFN pathway assay measurement	Clinical response to treatment	Association between measurement of IFN pathway activation and clinical response
**What is the evidence that interferon measurement is responsive (i.e. changes with changing disease status or treatment)?**				
	People presenting with any RMD	IFN pathway assay measurement	Change in serial IFN measurements	Responsiveness (change) of serial measurements of IFN pathway activation

Supplementary Text 2: Search Strategy for Ovid MEDLINE

Details of search strategy in Ovid MEDLINE(R) and Epub Ahead of Print, In-Process & Other Non-Indexed Citations and Daily <1946 to October 29, 2019>

1 interferon/ (21958)

2 (interferon* adj2 (biomarker* or sign*)).ti. (992)

3 (interferon* adj2 (biomarker* or sign*)).ab. /freq=2 (246)

4 exp interferon type i/ (48691)

5 "type 1 IFN".ti. (41)

6 "type 1 IFN".ab. /freq=2 (90)

7 (type 1 adj3 interferon*).ti. (205)

8 (type 1 adj3 interferon*).ab. /freq=2 (98)

9 (interferon* adj1 (alpha or alfa)).ti. (12678)

10 (interferon* adj1 (alpha or alfa)).ab. /freq=2 (5014)

11 (interferon* adj1 -beta).ti. (4226)

12 (interferon* adj1 beta).ab. /freq=2 (1557)

13 or/1-12 [inteferons] (71976)

14 Lupus Erythematosus, Systemic/ (53310)

15 (systemic adj2 lupus).ti. (28721)

16 (systemic adj2 lupus).ab. /freq=2 (4269)

17 exp Arthritis, Rheumatoid/ (110425)

18 ((reumat* or rheumat* or psoriatic or juvenile or inflammatory or idiopathic) adj3 (arthrit* or artrit*)).ti. (71763)

19 ((reumat* or rheumat* or psoriatic or juvenile or inflammatory or idiopathic) adj3 (arthrit* or artrit*)).ab. /freq=2 (21982)

20 Arthralgia/ (7938)

21 arthralgia.ti. (631)

22 arthralgia.ab. /freq=2 (888)

23 Connective Tissue Diseases/ (6410)

24 connective tissue disease*.ti. (3395)

25 connective tissue disease*.ab. /freq=2 (1408)

26 exp Scleroderma, Systemic/ (20043)

27 (scleroderma or systemic sclerosis).ti. (17190)

28 (scleroderma or systemic sclerosis).ab. /freq=2 (6774)

29 Sjogren's Syndrome/ (12463)

30 ((sjogren* or sjoegren or sicca) adj2 syndrome).ti. (9830)

31 ((sjogren* or sjoegren or sicca) adj2 syndrome).ab. /freq=2 (3362)

32 (spondyloarthropath* or spondylarthropath*).ti. (1514)

33 (spondyloarthropath* or spondylarthropath*).ab. /freq=2 (748)

34 Spondylitis, Ankylosing/ (14342)

35 ankylosing spondylitis.ti. (8361)

36 ankylosing spondylitis.ab. /freq=2 (2758)

37 Vasculitis/ (12667)

38 vasculitis.ti. (11783)

39 vasculitis.ab. /freq=2 (9137)

40 Antiphospholipid Syndrome/ (7889)

41 (antiphospholipid syndrome or APS or APLS).ti. (5112)

42 (antiphospholipid syndrome or APS or APLS).ab. /freq=2 (9483)

43 Still's Disease, Adult-Onset/ (1280)

44 Still's Disease.ti. (1536)

45 Still's Disease.ab. /freq=2 (311)

46 exp Myositis/ (19181)

47 myositis.ti. (4738)

48 myostitis.ab. /freq=2 (0)

49 (Behcet* adj (disease or syndrome)).ti. (7844)

50 (Behcet* adj (disease or syndrome)).ab. /freq=2 (2829)

51 ((IgG4* or Immunoglobulin G4*) adj2 (syndrome or disease)).ti. (1246)

52 ((IgG4* or Immunoglobulin G4*) adj2 (syndrome or disease)).ab. /freq=2 (1188)

53 or/14-52 [RMD] (291413)

54 13 and 53 (1855)

55 exp animals/ not humans.sh. (4641021)

56 54 not 55 (1754)

57 remove duplicates from 56 (1744)

58 limit 57 to english language (1563)

Supplementary Text 3: Search strategy for EMBASE

Details of search Strategy Embase Classic+Embase <1947 to 2019 October 30>

----

1 interferon/ (78376)

2 (interferon* adj2 (biomarker* or sign*)).ti. (1447)

3 (interferon* adj2 (biomarker* or sign*)).ab. /freq=2 (518)

4 alpha interferon/ (54088)

5 alpha interferon A/ (289)

6 beta interferon/ (24399)

7 (interferon* adj1 (alpha or alfa)).ti. (15287)

8 (interferon* adj1 (alpha or alfa)).ab. /freq=2 (6077)

9 (interferon* adj1 beta).ti. (5808)

10 (interferon* adj1 beta).ab. /freq=2 (2295)

11 (type 1 adj3 interferon*).ti. (350)

12 "type 1 INF*".ab. /freq=2 (86)

13 or/1-12 [type 1 interferon] (151212)

14 systemic lupus erythematosus/ (96577)

15 (systemic adj2 lupus).ti. (40830)

16 (systemic adj2 lupus).ab. /freq=2 (6638)

17 exp Arthritis, Rheumatoid/ (219451)

18 ((reumat* or rheumat* or psoriatic or juvenile or inflammatory or idiopathic) adj3 (arthrit* or artrit*)).ti. (114993)

19 ((reumat* or rheumat* or psoriatic or juvenile or inflammatory or idiopathic) adj3 (arthrit* or artrit*)).ab. /freq=2 (41573)

20 Arthralgia/ (60181)

21 arthralgia.ti. (911)

22 arthralgia.ab. /freq=2 (1866)

23 Connective Tissue Diseases/ (11630)

24 connective tissue disease*.ti. (4727)

25 connective tissue disease*.ab. /freq=2 (2429)

26 Scleroderma, Systemic/ (17314)

27 (scleroderma or systemic sclerosis).ti. (26554)

28 (scleroderma or systemic sclerosis).ab. /freq=2 (12470)

29 Sjogren's Syndrome/ (12089)

30 ((sjogren* or sjoegren or sicca) adj2 syndrome).ti. (13295)

31 (spondyloarthropath* or spondylarthropath*).ab. /freq=2 (1029)

32 Spondylitis, Ankylosing/ (15590)

33 ankylosing spondylitis.ti. (13053)

34 ankylosing spondylitis.ab. /freq=2 (5036)

35 Vasculitis/ (40212)

36 vasculitis.ti. (16629)

37 vasculitis.ab. /freq=2 (15699)

38 Antiphospholipid Syndrome/ (16255)

39 (antiphospholipid syndrome or APS or APLS).ti. (7161)

40 (antiphospholipid syndrome or APS or APLS).ab. /freq=2 (14445)

41 Still's Disease, Adult-Onset/ (1317)

42 still's disease.ti. (2021)

43 still's disease.ab. /freq=2 (519)

44 Myositis/ (16388)

45 myositis.ti. (77)

46 myositis.ab. /freq=2 (37)

47 Behcet Syndrome/ (8519)

48 (Behcet* adj2 (syndrome or disease)).ti. (11087)

49 (Behcet* adj2 (syndrome or disease)).ab. /freq=2 (4765)

50 ((IgG4* or "Immunoglobulin G") adj2 (sydrome or disease)).ti. (1569)

51 ((IgG4* or "Immunoglobulin G") adj2 (sydrome or disease)).ab. /freq=2 (1713)

52 or/14-51 [RMD] (523688)

53 13 and 52 [interferon type 1 and RMD] (9125)

54 (exp animal/ or nonhuman/) not exp human/ (7037850)

55 53 not 54 (8634)

Supplementary Text 4: Search strategy for Web of Science

Indexes=SCI-EXPANDED, CPCI-S, ESCI Timespan=1900-2019

1 (TS=((interferon* Near/1 (alpha or alfa)))) AND LANGUAGE: (English)

2 (TS=((interferon* Near/1 beta))) AND LANGUAGE: (English)

3 (TS=((type 1 Near/3 interferon*))) AND LANGUAGE: (English)

4 TS=("type 1 INF*") or TS=(interferon* Near/2 biomarker*) or TS=(interferon* Near/2 sign*)

5 #4 OR #3 OR #2 OR #1

6 TS=((systemic Near/2 lupus))

7 TS=(((reumat* or rheumat* or psoriatic or juvenile or inflammatory or idiopathic) Near/3 (arthrit* or artrit*)))

8 TS=(Arthralgia)

9 TS=("connective tissue disease*")

10 TS=((scleroderma or "systemic sclerosis"))

11 TS=(((sjogren* or sjoegren or Sjogren's or sicca) Near/2 syndrome))

12 TS=((spondyloarthropath* or spondylarthropath*))

13 TS=("ankylosing spondylitis")

14 TS=(vasculitis)

15 TS=(("antiphospholipid syndrome" or APS or APLS))

16 TS=("still's disease")

17 TS=(Myositis)

18 TS=((Behcet* Near/2 (syndrome or disease)))

19 TS=(((IgG4* or "Immunoglobulin G") Near/2 (sydrome or disease)))

20 #19 OR #18 OR #17 OR #16 OR #15 OR #14 OR #13 OR #12 OR #11 OR #10 OR #9 OR #8 OR #7 OR #6

21 (#20 AND #5) AND LANGUAGE: (English)

22 (TS=("type 1 INF*") or TS=(interferon* Near/2 biomarker*) or TS=(interferon* Near/2 sign*)) AND LANGUAGE: (English)

24 (#23 AND #20) AND LANGUAGE: (English)

23 #22 OR #3 OR #2 OR #1

Supplementary Text 6: Inclusion and exclusion criteria

**Inclusion criteria**
Subjects: human patients with RMDs<br><br>RMDs: systemic lupus erythematosus (SLE), rheumatoid arthritis (RA), arthritis, juvenile idiopathic arthritis (JIA), arthralgia, Sjögren's syndrome (pSS), Sicca syndrome, dermatomyositis (DM), polymyositis (PM), myositis, connective tissue disease (CTD), scleroderma, systemic sclerosis (SSc), psoriatic arthritis (PsA), ankylosing spondylitis (AS), spondyloarthropathies (SpA), vasculitis, NCA-associated vasculitis (AAV), giant cell arteritis (GCA), antiphospholipid syndrome (APS), Still disease, adult-onset Still disease (AOSD), Behçet disease (BD), IgG4-related disease (IGRD)<br><br>Language: English only<br><br>Study design: longitudinal studies, cross-sectional studies, randomized controlled trials, case-control studies, cohort studies, non-controlled trials, intervention studies<br><br>Samples: blood, serum, plasma studies
**Exclusion criteria**
Subjects: animal studies, pre-clinical studies, genetic studies<br><br>Study design: case studies, letters, non-original articles (reviews, editorials, opinion pieces, etc)<br><br>Samples: peripheral tissues or fluids other than blood, serum or plasma<br><br>Papers that do not specify the type of interferon that the assay measures

Supplementary Text 6: Detailed description of assay methodology

Immunoassays: IFN-I protein

Immunoassays are biochemical methods based on the principle of antigen-antibody reaction which measures molecule of interest in suspension, here specifically type I IFNs such as IFN-α or IFN-β. To measure IFNs levels in serum or plasma samples from RMD patients' immunoassays in many different formats and variations were used with IFN-α being evaluated in n=58 studies and IFN-β n=9. Although sharing same principle of measurements, immunoassays varied in protein capture and readouts. Assays used mostly rely on commercially available platforms such as Enzyme linked immunoassays (ELISAs) and Flow cytometry based assays such as LUMINEX or cytometric bead array (CBA), radioimmunoassay (RIA), dissociation-enhanced lanthanide fluorescence immunoassay (DELFIA), elecrochemiluminescence by Meso Scale Discovery (MSD) or other multiplex platforms. A number of the papers did not specify what subtype of IFN-α particular assay was measuring while majority are based on measuring IFN-α2 (commercially available IFNα2a, IFNα2b, and IFNα2c) as the most potent subtype of IFN-α. In rare cases a more detailed description was provided [21].

ELISA is the most widely reported method for measuring a single cytokine in biomedical research and clinical laboratory testing and was the most frequent in the IFN literature. Most studies reported single molecule ELISAs, with multiplex assays that captured IFN-I as part of a panel of cytokines and chemokines, or other soluble proteins were reported in some cases. Multiplexing was used by several methods described below and it is particularly useful if volume of biological sample is limited and one wants to measure as many analytes as possible. Most of the assay performance data provided was based on manufacturer literature. In papers using ELISAs some showed a lack of sensitivity for IFN-I with levels in healthy range being reported as undetectable [62, 124]. Such low levels of circulating IFN protein potentially reflect its' high biological potency and also high uptake by numerous cells because most cell types express type I IFN receptor, but also non-circulating sources. The dynamic range of ELISA-based assays is narrower than that of other technologies such as multiplex assays. Sample dilution is often required for the assay and consequently creates variations of cytokine level between the neat samples and diluted samples.

DELFIA is a time-resolved fluorescence assay and is an alternative to classical ELISA which does not use a colorimetric enzyme. Instead, reagents are labelled with Europium which dissociates once the antibody-analyte immune rection is complete and the fluorescence is measured by time-resolved fluorometry (TRF). Depending on the antibodies used, this method may detect most IFN-α subtypes but not always IFN-α2b. DELFIA assay to measure IFNs was used in n=8 reports and only to measure IFN-α protein. None reported DELFIA for other IFN-I.

Luminex assays capture targets in suspension onto spherical color-coded beads, pre-coated with analyte-specific capture antibodies and enables multiple proteins to be measured at the same time. Luminex is faster and more efficient, detects analytes in a broad dynamic range of concentrations. These assays are flow cytometry-based, which also conveniently allows multiplexing however here each bead is coated with antibodies against different analytes and can be multiplexed to form assays measuring multiple

proteins in the same samples. Limited information given in material and methods does not allow to give precise numbers of each of these methods used.

Assays using radiolabeled tracers (mostly I^{125}) were also used such as radioimmunoassay (RIA) and immunoradiometric assay (IRMA) in n=5 reports (n=3 RIA and n=2 IRMA). Both are very sensitive and specific *in vitro* techniques.

Single Molecule Array (SiMoA)
SiMoA is a recent technique that is orders of magnitude more sensitive than standard sandwich-based immunoassay methods. ELISA methods typically detect concentrations in the order of pg/ml, while SiMoA can achieve sensitivity as low as femtogram (fg/ml). This may be particularly important for IFN-I since these cytokines occur at low concentrations in biological samples such as serum. SiMoA therefore permits detection and quantification of these proteins at concentrations previously difficult or impossible to measure.

SiMoA achieves this increased sensitivity by capturing single molecules in femtoliter-sized wells, allowing for a “digital” readout of each individual bead to determine if it is bound to the target analyte or not. It has also low level of background signal that can be realized by the digitization of protein detection.

Immunoassays: IFN-inducible proteins
These assays measure IFN-inducible proteins that are secreted into the serum, plasma or remain intracellular (and may be measured in cell lysates, e.g. MxA). These are proteins known to be coded by IFN-stimulated genes such as SIGLEC-1, IFI16, MX1, and many chemokines such as CXCL9 and CXCL10. There were 42 reports on chemokines an IFN induced proteins (27 measured serum, 6 used plasma, 2 serum and/or plasma and 3 whole blood lysates). The literature contains a wide variety of different IFN induced soluble proteins, with several different combinations (**Figure 4** UpSet plot chemokines). In addition to the studies in this figure, one paper analysed more than 96 proteins. The choice of proteins is decided by the investigator and there was little consensus between studies. The most commonly measured proteins were CXCL10, CXCL11, CXCL9, CCL4 and CCL3 which were each measured in 9 or more studies. Combinations of proteins were not repeated often in the literature. The combination of CXCL10 and CCL19 was reported in four studies. A further four reported CXCL10 alone. 18 / 42 studies reported a unique protein or combination of proteins.

Flow Cytometry
Flow cytometry allows measurement of markers on specific subsets of cells without cell sorting. This may be advantageous compared to methods that analyse whole blood or unsorted PBMCs as it prevents artefactual changes secondary to changes in the size of cell populations. The method is limited by the interferon-stimulated proteins that are expressed on the cell surface or intracellular, and results will be dependent on the choice of antibody and fluorochrome used and gating strategy for the subset of interest. Methods in flow cytometry require standardization to harmonise between centres, which is particularly important when assessing fluorescence intensity.

Gene expression: RNA Microarrays
A microarray (or chip) is a flat surface on which 10,000–100,000 distinct oligonucleotide

probes (synthetic sequences) are bound, so it can only provide information about the transcripts that are included on the array. These probes represent unique sequences for individual genes that will allow for complementary binding of mRNA from cell/tissue samples. Hence this technique allows to measure the expression of pre-defined set of probes (transcripts). Most arrays are designed to cover only a portion of protein-coding genes, and do not evaluate regulation of non-coding genes (i.e., lncRNAs, pseudogenes), other novel RNAs or splice variants in a single array. After adding cDNA derived from patient samples, the transcript-specific sequence binds on the chip or hybridizes with the synthetic sequence. This method presents advantages over other transcriptomic methods like RNA-sequencing, such as faster computation time, smaller file sizes and more straightforward analysis. Depending on the amount of transcriptional change in the experiment, processing and summarizing of differentially expressed genes from a set of microarray-generated gene expression data can be completed within a few hours with user-friendly software.

Gene expression: RNA-sequencing

RNA sequencing allows for full sequencing of the whole transcriptome and can qualitatively and quantitatively investigate any RNA type including messenger RNAs (mRNAs), non-coding transcripts (i.e. microRNAs, small interfering RNAs, and long noncoding RNAs, pseudogenes), splice variants. Therefore, it may provide additional, informative data for prediction, mechanistic investigations or biomarker discovery over microarrays. The most common differential gene expression protocols use Illumina next generation sequencing systems. From an RNA sample, mRNA is selected by its polyadenylation tail and fragmented in preparation for Illumina sequencing, which produces “short reads” of hundreds of bases. Prior to this the mRNA is reverse transcribed into a library of complementary DNA (cDNA) for sequencing. Sequence reads are counted and mapped to a reference transcriptome. RNASeq has wider dynamic range than microarrays, and is able to identify a larger number of differentially expressed genes. Because of these advantages, RNA-Seq is progressively replacing microarray technology for many transcriptomic applications. It has also a few disadvantages compared to microarrays. RNA-Seq files are considerably larger (generally about >50 times larger than microarray files) depending on the sample size and sequencing depth which requires appropriate servers for data storage. Analysis is more complex requiring a bioinformatics expert to run a multiple bioinformatics tools. More intensive and expensive computation infrastructure and analytics, often using cloud-based servers may be used. Analysis may take longer than micro-arrays. However, these limitations are gradually improving.

Gene expression: Nanostring

The NanoString system hybridizes two probes to each target transcript: a biotin-labeled capture probe and a fluorescent barcode-labeled reporter probe. These are custom-made or pre-designed sets of two color-coded probes of ~50 base pairs per mRNA that hybridize in solution pre-mixed with a set of system controls. The reporter probes hybridize with specific RNAs in a sample and the capture probes lock them via avidin onto a static surface. The NanoString nCounter Analysis System counts the immobilized RNAs using their barcodes. No reverse transcription to produce cDNA is required. Like other hybridization-based systems, NanoString reports targeted transcriptomics (usually 800 target transcripts) rather than unbiased results for discovery science. The NanoString system can identify RNAs in a heterogeneous sample, even one that

contains cells from different species. The accurate design of probes allows to study interactions between hosts and pathogens, hosts and microbiomes, or tumour cells and the immune cells that respond to them at the transcript-level in a single sample. The nCounter has the capacity to input 12 samples and detect 800 genes.

Gene expression: IFN-I Scores by quantitative PCR

Quantitative PCR (qPCR) is used to quantify mRNA transcripts. The technique requires extraction of RNA from cellular material. This could be whole blood (collected into PAXgene n=46 studies or Tempus tubes to preserve RNA integrity in transport and long-term storage), PBMCs or sorted cell subsets (i.e. monocytes). A number of different platforms are then used to measure expression of ISGs, the most common using commercially available probes and primers with Taqman or Sybr-green, but some used probes designed by investigators. Studies varied in their number and choice of ISGs, their choice of reference genes, the method of calculating a summary, analyses performed and reporting practices.

DNA Methylation Assays

DNA methylation occurs when a methyl group is added to the fifth carbon of cytosine residues that are linked by a phosphate group to a guanine nucleotide (a CpG dinucleotide) by DNA methyltransferases. Analysis of DNA methylation provides an indication of which genes are being actively transcribed. In general, hypomethylation of promotor regions leads to increased transcription. DNA methylation can be analysed for both ISGs and IFNs themselves.

To analyse DNA methylation, bisulfite-treated genomic DNA is mixed with assay oligonucleotides, one of which is complementary to uracil (converted from original unmethylated cytosine), and another that is complementary to the cytosine of the methylated (and therefore protected from conversion) site. Following hybridization, primers are extended and ligated to locus-specific oligonucleotides to create a template for universal PCR. Finally, labelled PCR primers are used to create detectable products that are immobilized to bar-coded beads, and the signal is measured. The ratio between two types of beads for each locus (individual CpG) is an indicator of its methylation level. In usual settings, the main goal for methylation testing is to find CpG sites that are differentially methylated between two groups of samples, in order for example, to identify diagnostic biomarkers for diseases such as SLE and others.

Reporter-cell Assays

Reporter cell assays also called Reporter gene assays (RGA) allows quantitative analysis of the ability of IFNs in a serum or plasma sample to upregulate expression of number of ISGs by reporter cells, subsequently measured by qPCR. The most commonly used cell lines in RGA are HeLa or HeLa-derived cells (*i.e.* Wish). Such assays are measured using expression of ISGs, and therefore some of the comments under gene expression assays, such as choice of ISGs, may also apply.

Other assays use Vero cells transfected with a plasmid carrying the luciferase gene under the control of a type I IFN inducible promoter further analysed by luminometer. These assays therefore measure the protein encoded by a single ISG.

Additionally, cell lines like RAW-Blue ISG and B16-Blue IFNα/β have been recently

engineered. Upon stimulation with IFN from a sample, these cells produce a soluble gene product (i.e. secrete ALP) that can be quantitated using microplate spectrophotometers or luminometers.

Cytopathic Effect Assays

This method measures the capability of IFN-I in the serum or plasma of a patient to suppress the cytopathic effect (CPE) on a target cell induced by an infectious viral replication. It therefore measures IFN bioactivity as protection of a cell line from viral challenge by IFN-I. The cytopathic effect refers to morphological or structural changes in host cells that are caused by cytopathogenic virus invasion. These include rounding of cells, forming inclusion bodies in cell nucleus or cytoplasm of the host cells, formation of scynthia (large fused cellular masses that contain many nuclei). These effects are usually evaluated by microscopy, although more recently colorimetric and spectrophotometric readouts have been used. The results of these assays are defined as defined as the quantity of IFN-I that reduces the CPE of viral infection by 50%. A standard curve using known recombinant IFN dilutions, as well as serial dilutions of the sample, are needed to allow qualitative or semiquantitative results. Many researchers used the human lung carcinoma cell line A549 due to their ease of growth and relatively high sensitivity to all forms of human IFNs. These cells are usually challenged with EMCV. Human IFN-α can be assayed using the bovine kidney cell line MDBK challenged with VSV. One advantage of this system is the relative insensitivity of the bovine cells to human IFN-β and -γ giving some selectivity in uncharacterized samples. Human IFN-β can also be measured on the green monkey kidney cell line Vero challenged with VSV. Human IFN-α is much less active on these cells than IFN-β.

Plaque Reduction Assay

Plaque reduction assays remain one of the most accurate methods for the direct quantification of infectious virons multiplying in cells in virology. The applications in virology are diverse, but among them, these assays also allow the detection of antiviral substances including IFNs. The effect of an antiviral substance is inferred through the counting of formed plaques in cell culture, which can show infectious units, cellular dead zones, circular zones of infected regions and plaques. When the assay is used to measure IFN-I in a patient sample, the patient sample is included alongside a virus and cell line and then the plaque reduction neutralization test (PRNT) is evaluated.

A preselected cell line is grown in confluent monolayer with presence of serially diluted patient serum / plasma or controls. Further culture is infected with a lytic virus. Individual plaques will begin to develop as viral infection and replication are constrained by presence of IFN-I in the patient sample to the surrounding monolayer. Infected cells will continue the replication-lysis-infection cycle, further propagating the infection, resulting in increasingly distinct and discrete plaques. Depending on the viral growth kinetics and host cell used, a visible plaque will normally form within 2-14 days. Plaques may then be counted with a standard bright field microscope, or more typically fixed and counterstained by neutral red or crystal violent in order to be readily identified by naked eye. Antiviral effect is then quantifed as plaque forming units per milliliter. Alternatively, neutralizing capacity of the sample is determined by calculating the percentage reduction in total virus infectivity by counting number of plaques and given as highest dilution that reduced the number of virus plaques by 50%. Depending on the visualization method,

plaques can be counted immediately or later (in the case of immunostaining or cell monolayer coloration). A log drop should be noted between serial dilutions, with a negative control used as a reference. Samples need to be tested in replicates.

The ability of plaque assays to accurately assess viral titers may be influenced by numerous factors: host cell selection, media and growth conditions for cellular and viral viability, sufficient serum dilution ranges in order to clearly differentiate plaques, immobilized viral propagation, and an accurate determination of the viral incubation period to allow adequate time for distinct and countable plaque formation. Plaque morphology can vary dramatically under differing growth conditions and between viral species used. Internal quality controls therefore need to be included.
